# Technology-Enabled Mental Health Service Reform for Open Arms – Veterans and Families Counselling: Participatory Design Study

**DOI:** 10.2196/13662

**Published:** 2019-09-19

**Authors:** Haley M LaMonica, Tracey A Davenport, Jane Burns, Shane Cross, Stephanie Hodson, Jennifer Veitch, Ian B Hickie

**Affiliations:** 1 Brain and Mind Centre The University of Sydney Camperdown Australia; 2 InnoWell Pty Ltd Camperdown Australia; 3 Faculty of Health Sciences The University of Sydney Camperdown Australia; 4 Open Arms-Veterans & Families Counselling Canberra Australia

**Keywords:** veterans, mental health, technology, community-based participatory research, health care reform, stakeholder participation

## Abstract

**Background:**

The impact of mental ill-health on every aspect of the lives of a large number of Australian Defence Force (ADF) personnel, their partners, and their families is widely recognized. Recent Senate inquiries have highlighted gaps in service delivery as well as the need for service reform to ensure appropriate care options for individuals who are currently engaged with mental health and support services as well as for those who, for a variety of reasons, have not sought help. To that end, successive Australian governments generally and the Department of Veterans’ Affairs specifically have prioritized veteran-centric reform. Open Arms is an Australia-wide service that provides counseling and support to current and former ADF personnel, and their family members, for mental health conditions.

**Objective:**

The aim of this study was to develop and configure a prototypic Web-based platform for Open Arms – Veterans & Families Counselling (formerly Veterans and Veterans Families Counselling Service) with the Open Arms community to enhance the quality of mental health services provided by Open Arms.

**Methods:**

The study aimed to recruit up to 100 people from the Open Arms community (current and former ADF personnel and their families, health professionals, service managers, and administrators) in regions of New South Wales, including Sydney, Canberra, Maitland, Singleton, and Port Stephens. Participants were invited to participate in 4-hour participatory design workshops. A variety of methods were used within the workshops, including prompted discussion, review of working prototypes, creation of descriptive artifacts, and group-based development of user journeys.

**Results:**

Seven participatory design workshops were held, including a total of 49 participants. Participants highlighted that the prototype has the potential to (1) provide the opportunity for greater and better-informed personal choice in relation to options for care based on the level of need and personal preferences; (2) ensure transparency in care by providing the individual with access to all of their personal health information; and (3) improve collaborative care and care continuity by allowing information to be shared securely with current and future providers.

**Conclusions:**

Our findings highlight the value of actively engaging stakeholders in participatory design processes for the development and configuration of new technologies.

## Introduction

### Veteran-Centric Reform

Successive Australian governments have been concerned about the mental health and well-being of current and former Australian Defence Force (ADF) personnel and their families and have committed to improve policies and services to prevent and treat mental health conditions and support individuals and families in need [1]. However, recent government inquiries concerning the mental health of the ADF [2] and suicide by current and former personnel [3] have revealed the enormous impact of mental ill-health on every aspect of the lives of a large number of ADF personnel, their partners, and their families. The inquiries highlighted gaps in service delivery as well as the need for service reform and also strategies and services that can appropriately deal both with those currently engaged with mental health and support services and those who, for a variety of reasons, have not sought help [1,2].

In March 2015, the Department of Veterans’ Affairs (DVA), including services provided by Open Arms – Veterans & Families Counselling (formerly Veterans and Veterans Families Counselling Service; renamed October 2018), was supporting 49,668 veterans who had 1 or more mental health disabilities [4]. In light of the service gaps noted in the recent Senate Inquiry reports, DVA has prioritized veteran-centric reform [5] to ensure dramatic improvements in the way DVA connects with veterans and to provide the former service community with a greater standard of service through business processes, culture, and government-endorsed best practice mental health service delivery.

Regarding access to mental health and support services for current and former ADF personnel, the 2017 Senate Inquiry report highlighted the vital role of Open Arms as a trusted provider of high-quality mental health services for the Defence community [3]. Open Arms is an Australia-wide service that provides counseling and support to current and former ADF personnel, and their family members, for mental health conditions (such as posttraumatic stress disorder, anxiety, depression, sleep disturbance, and anger). Open Arms also provides relationship and family counseling. In 2017, Open Arms provided support to 27,000 current and former ADF personnel and their families [6]. On the basis of the outcomes of an independent Service Functional Review commissioned by DVA in 2014 [7], Open Arms has identified a number of critical challenges related to service quality, including prevention and early identification, fast tracking support, need for engagement outside the health care system, capacity to provide the right care at the right time, and complex psychosocial needs of persons accessing the service.

### Technology Solutions for Mental Health Services Reform

In June 2017, the University of Sydney’s Brain and Mind Centre and DVA partnered to engage the Open Arms client base (current and former ADF personnel and their family members) and staff (health professionals, service managers, and administrators) in participatory design workshops to explore how a prototypic Web-based platform could be tailored to realize a technology-enabled solution for the Open Arms service.

Originally, the prototype was designed, built, and evaluated through a partnership between the Young and Well Cooperative Research Centre and the University of Sydney’s Brain and Mind Centre [8] known as Project Synergy. Project Synergy (Phase I: 2014-2016) was originally commissioned by the Australian Government Department of Health (DOH) in 2014, with the broad aim of transforming the provision of mental health care across Australia by harnessing the potential of new and emerging technologies to reach all people, regardless of location, and provide them with access to timely and evidence-based treatment to improve their mental health and well-being. The Funding Agreement provided for the establishment of a Research and Development (R&D) Group as well as a Product Group for the development of the prototypic Web-based platform.

This prototype links integrated and interoperable resources (eg, apps, etools, and Web-based and in-clinic health services, most with data-sharing functionality) to enhance service quality; track real-time health and social outcomes; and bring integrated, high-quality, and personalized service experiences to the person seeking care. It operates through existing health providers, such as Open Arms, to promote access to high-quality and cost-effective mental health services.

Importantly, the goal of the prototype is to offer immediate Web-based assessment (all individuals complete a tailored self-report questionnaire) resulting in a personalized dashboard of results. The results give individuals an overall profile of their health and well-being (including mental health), which can be shared with their health professional, other health care providers, and family members, among others (dependent on permission being granted by the individual). The prototype uses staged care on the basis of the transdiagnostic clinical staging model [9,10] to identify the extent of progression of disease at a point in time. This enables the platform to match recommendations including apps, etools, and clinical interventions to an individual’s level of need.

At its core, the prototype promotes person-centered health care and its principles highlight that individual clients of a service are equal partners in their health care. To that end, to promote transparency, individuals have access to all information that directly concerns them. Furthermore, all information is presented in plain language (including avoiding jargon), and individuals are presented with sufficient information to understand all components of the prototype (eg, initial self-report questionnaire, dashboard of results), with options for obtaining further information if desired. Critically, decisions about an individual’s care are made collaboratively with a health professional or service, taking into account both clinical needs and personal preferences. The prototype helps minimize variability in care provision between individual health professionals and services by using evidence and data rather than solely relying on clinical opinion, which can be variable and fallible. Finally, the prototype is being designed to maximize the use of resources and minimize the duplication of services and wastage of time, for all individuals.

A key feature of the prototype is that it is able to be configured to meet the needs of all end users, including individuals and supportive others, health professionals, service managers, and administrators. By engaging potential end users through the iterative use of participatory design, the prototype can be continuously redeveloped to best meet the needs of a health care service.

Research has shown that the active participation of all stakeholders throughout the design of technical systems and services helps ensure that the end product meets the needs of its intended user base, improves usability, and increases engagement of all individuals [11,12]. Through the engagement of stakeholders in participatory design, technical solutions to practical problems related to health care are generated as a means to effect organizational (ie, service) reform [13]. Importantly, end users (in this instance, all members of the Open Arms community) have the opportunity to actively co-design the technology solution in conjunction with researchers and product designers with the aim of developing Web-based tools and systems that are more likely to be engaging and effective for all users [11,14].

The aim of the current research was to actively engage individuals from the Open Arms community, via participatory design, to collaboratively develop and configure the prototype to enhance Open Arms service quality.

## Methods

### Ethics

The research study was approved by the DVA Human Research Ethics Committee (project number: E016/027).

### Participants

The study aimed to recruit up to 100 people from the Open Arms community, including current and former ADF personnel and their families, as well as health professionals (including Open Arms counselors and Outreach Program Counselors [OPCs]), service managers, and administrators in regions of New South Wales, including Sydney, Canberra, Maitland, Singleton, and Port Stephens.

Inclusion criteria for current and former ADF personnel and their families were as follows: aged 16 years or older and eligible for Open Arms services (all current and former ADF personnel who have at least 1 day continuous full-time service are eligible for care through Open Arms; having met this criterion, their family members are also eligible for care).

Representatives from the Australian Government, including senior executives in the DOH, DVA, and Open Arms, as well as members of Project Synergy’s R&D and Product Groups, were also included in the participatory design workshops as key stakeholders; however, the data presented in this paper reflect the population (ie, current and former ADF personnel and Open Arms health professionals, service managers, and administrators).

### Recruitment Strategy

To recruit current and former ADF personnel and their families, the recruitment strategy included the distribution of postcards and A3/A4 posters with information about the participatory design workshops in the lead up to each scheduled workshop in each of the 5 regions. Targeted social media (location matched to each relevant region and age more than 16 years) was also used to circulate information about the workshops. To avoid any perceived coercion, recruitment was passive such that a potential participant needed to contact the Research Project Manager who, only upon a potential participant’s request, then forwarded the Study Information Sheet and Participant Consent Form.

For the recruitment of health professionals (including Open Arms counselors and OPCs), service managers, and administrators, information was distributed to potential participants directly by the relevant Open Arms Centre managers in each of the 5 regions.

All participants were provided with detailed information about the study both before attending a participatory design workshop and upon arrival at the workshop. At the beginning of each workshop, the facilitators provided the participants with an opportunity to ask questions and clarify details of the research before providing written informed consent. Potential participants were reminded that participation was entirely voluntary, and that if they agreed to participate, they could withdraw their consent at any time without being required to provide any reasons and with no impact on their relationship with Open Arms or the University of Sydney’s Brain and Mind Centre.

### Participatory Design Workshops

The workshops brought together members of the Open Arms community, Australian Government representatives, and representatives from Project Synergy’s R&D and Product Groups to explore how the prototype could be developed and configured to enhance mental health services provided by Open Arms. All workshops were coordinated by at least 2 facilitators, 1 of whom was a mental health professional whose role was to respond to any participant concerns or distress as a result of the subject matter. An additional Open Arms counselor was also present for this purpose. A scribe was present to take handwritten notes throughout the workshop.

Each workshop (4-hour duration) was designed to actively engage participants in interactive discussions about how to co-design potential technology solutions for the Open Arms service. The facilitators used a variety of methods within the workshops, including prompted discussion, review of working prototypes (wireframes), creation of descriptive artifacts, and group-based development of user journeys (a series of steps illustrating how an individual might interact with the prototype). Importantly, user journeys help to understand user behavior and identify other possible functionality at a high level and define taxonomy and interface. They refer to personas and real people and feedback into a number of technology-building activities including information architecture and sitemaps, the development of wireframes, and functional specifications.

To ensure maximum coverage of a number of critical issues to develop the technology solution, several areas of focus were explored, including: the intake; triage and waitlist processes at Open Arms; typical pathways to care at Open Arms; expectations of care provided by Open Arms; and potential for technology use for health and well-being as well as health-related emergencies. The above-listed topics reflected potential areas for service reform identified through an independent functional review of Open Arms commissioned by DVA in 2013-2014 [7]. Through weekly meetings, the agenda items and methods were then collaboratively refined by the joint Project Management Team, comprising researchers, health professionals, and members of Open Arms to determine how best to discover how the technology-enabled solution might enhance or reform Open Arms for improved outcomes for clients and their families.

### Data Analysis

At the conclusion of each workshop, the notes taken by the scribe were transcribed into a report documenting the participant profile (ie, gender and participant type such as health professional or veteran) as well as the content of the discussion relative to the agenda. These reports in combination with the visual artifacts collected during the participatory design workshops (nonidentified and presented in aggregate to ensure confidentiality) were analyzed by members of the R&D Group using knowledge translation processes to identify themes and key learnings and inform the development and configuration of the prototype for the Open Arms service, particularly in relation to improved service quality.

Knowledge translation is defined as the synthesis, exchange, and application of knowledge by stakeholders to enhance the benefits of innovation in strengthening health systems and improving health outcomes [15]. Broadly, knowledge translation promotes translation of research findings into clinical practice, organizational management, technology development, and policy reform, bridging what has been coined *the know-do gap* [15]. It is an interactive process between researchers and health care systems to pinpoint R&D priorities that will benefit a service, including clients, health care professionals, and administrative personnel [16].

The analysis was guided by research questions related to the critical challenges identified in the Service Functional Review [7], including elements of the Open Arms service pathway, such as intake, assessment, and treatment. Categories relating to required prototype functionality (including data security and confidentiality) were also explored as they were likely to critically inform development and configuration. Using the above identified areas of interest as a guide, 2 researchers (HL and CR) independently analyzed the data. In accordance with knowledge translation processes previously used by our group [17], observations were tallied, and those observations with 3 or more independent tallies were considered to be consistent themes. Observations included comments by individual participants (eg, current and former ADF personnel and their families as well as Open Arms health professionals, service managers, and administrators) as well as comments generated during small group discussions, including when developing user journeys or prototypes. As the participatory design workshop agendas varied, building on the learnings from each previous session, consistency in themes across workshops was not examined. Although members of the Project Synergy R&D and Product Groups were active participants in the workshops to drive the collaborative development and co-design process, their comments were not included in the analyses.

The primary researchers came to a consensus on any themes that were not agreed upon during the independent analyses. A third independent researcher (TD) subsequently checked the themes against the available data and in relation to the research questions.

The quotes referenced in the paper are representative of all participant types (ie, current and former ADF personnel and their families, health professionals, service managers, and administrators); however, as the majority of the quotes were derived from small, mixed group discussions (ie, a spokesperson relaying the views of a group) and are not reflective of a single individual’s or participant type’s point of view, coding has not been used within the paper.

## Results

### Demographics

A total of 7 participatory design workshops (all of 4-hour duration) were held between August and September of 2017. The aim of each workshop was to actively engage the Open Arms community in discussions about how to collaboratively develop and configure technology solutions aimed at enhancing the Open Arms service.

A total of 49 Open Arms–recruited participants attended the workshops, comprising a mix of participants from the Open Arms community, including current and former ADF personnel and their families as well as Open Arms health professionals, service managers, and administrators (Table 1). No participants expressed concern or experienced any distress in any of the workshops.

**Table 1:** Participant demographics (N=49).

Demographic		Statistics
**Participants**		
	Female, n (%)	28 (57)
**Role^a^, n**		
	Former personnel	13
	Current serving personnel	6
	Family of current or former personnel	10
	Health professional	17
	Representative from Australian Government Department of Health	9

^a^Some participants fulfilled multiple roles (eg, ex-serving personnel and health professional).

The results presented below highlight the consistent themes identified by participants in relation to the potential impact of the prototype for Open Arms clients and the service pathway, particularly in relation to the intake process, provision of personalized recommendations matched to level of need, and routine progress monitoring. In addition, possible technology solutions to improve risk detection and suicide prevention were evaluated.

### Principles of the Prototype for the Open Arms Service

#### Personal Choice

Participants highlighted the potential for the prototype to promote personal choice among clients of the Open Arms service:

[The prototype] could support clients to make choices on intake about service engagement and options for care.

Clients should be able to make their own choices about what aspects of their health and wellbeing is most important for them to work on.

It is important to provide the client with the choice to share his or her data with a counsellor.

#### Transparency

In particular, participants noted that critical information should be provided to ensure informed decision making and transparency (eg, limitations to confidentiality, details about the assessment and reporting processes, clear terms and conditions, and background information about how recommendations have been generated):

[The prototype] should be straightforward and transparent. For example, the [prototype] needs to provide details about the length of any and all assessments, how the suggested apps are rated, and how the dashboard is generated.

Data security needs to be highlighted, particularly that the information is not shared with DVA.

#### Collaborative Decision Making

Participants supported the concept of shared treatment planning, frequently noting that this approach to care would promote engagement, adherence, and communication:

[The prototype] will enable shared treatment planning, giving the client ownership over their care.

The Shared Care Plan will promote engagement and facilitate communication between the client and health professional.

#### Continuity of Care

Participants agreed that a prototype that eliminates the need for an individual to have to repeat *their story* to multiple care providers would be highly valued by the Open Arms community:

[The prototype] will limit the need for people to have to tell their story over and over again.

The process of repeatedly writing out your story to each service is extremely traumatic. To have the option of having it written down, or already existing within a [prototype], would reduce the distress associated with this process.

Veterans want to avoid telling their story multiple times. Data and information should be stored in [the prototype], ready to be shared with another service, if needed.

For individuals with a history of trauma, repeating their story is particularly upsetting.

#### Staged Care

Participants recognized the potential benefits of employing a staging model in the prototype to align recommendations (eg, apps, etools, and clinical interventions) with an individual’s current level of need:

Clinical services can be matched to the degree of need. It will be important to highlight that the services provided are evidence-based solutions.

Just because someone does an intake, doesn’t mean they need to use the service. Providing the individual with psychoeducation and self-management techniques could be appropriate.

#### Configuration of Content for the Open Arms Community

Participants consistently noted that the design and content of the prototype needs to be specific to the veteran community in recognition of their unique experiences and needs from a health care service:

The importance of the need to customise tone, language and aesthetic to the individual based on their age, relationship to the military and/ or to the veteran was emphasised.

Anything that is offered to [veterans] needs to be seen to have some level of veteran specificity.

#### Plain English

The need to provide all information to individuals in a clear and straightforward manner was emphasized by participants:

It is imperative to avoid clinical jargon. The language needs to be geared towards the veteran community.

### Open Arms Service Pathway Themes

#### Intake

**Figure 1:**
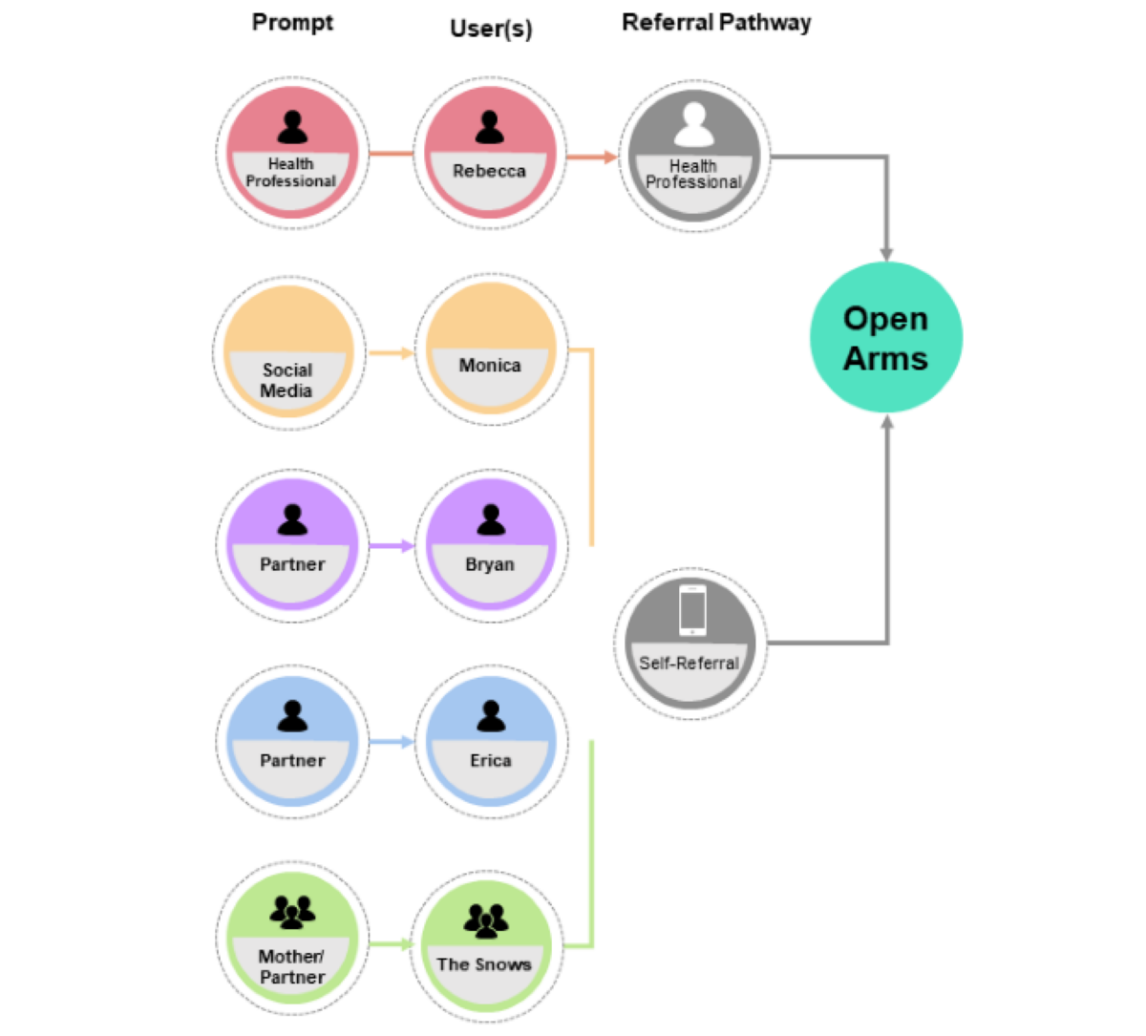
Possible points of Open Arms service entry based on user journeys.

As part of 4 of the participatory design workshops, participants jointly created user journeys to characterize how a client might engage in care with Open Arms, through different points of intake depending on their persona (Figure 1). As highlighted (Figure 1), an individual may be prompted to make contact with Open Arms for the first time in several ways including by a health professional, social media, their partner, or other family member; however, participants reported that most frequently, a significant other, or family member, prompts the initial contact with Open Arms.

The participants made several suggestions for how the prototype could improve or support the Open Arms intake process, which, at the time this research was conducted, included a pre-enrollment screening, an intake assessment, allocation to a health professional, and appointment scheduling:

[The prototype] could help with triage, fast-tracking clients when needed (eg, if a client scores above a pre-specified threshold on a psychometric instruments, the [prototype] could escalate/ prioritise them for an appointment or contact their health professional).

When the initial online assessment is completed, [the prototype] could use that assessment to direct the client to a health professional with the specific expertise required or whom is most suitable.

Online intake process may also make services more accessible as it may be a way to engage individuals in care who are fearful of coming in to a physical [Open Arms] Centre.

**Figure 2:**
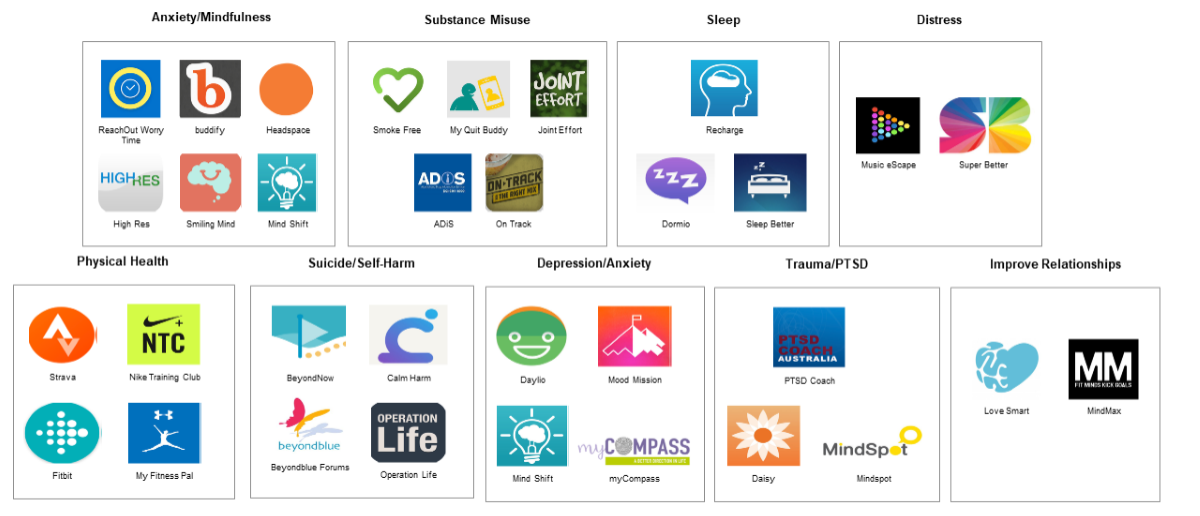
Recommendations based on an individual’s personalized dashboard of results.

Furthermore, participants recognized that the prototype could provide resources to individuals (such as recommended apps and etools) while they were on a waitlist to see a health professional (Figure 2).

**Figure 3:**
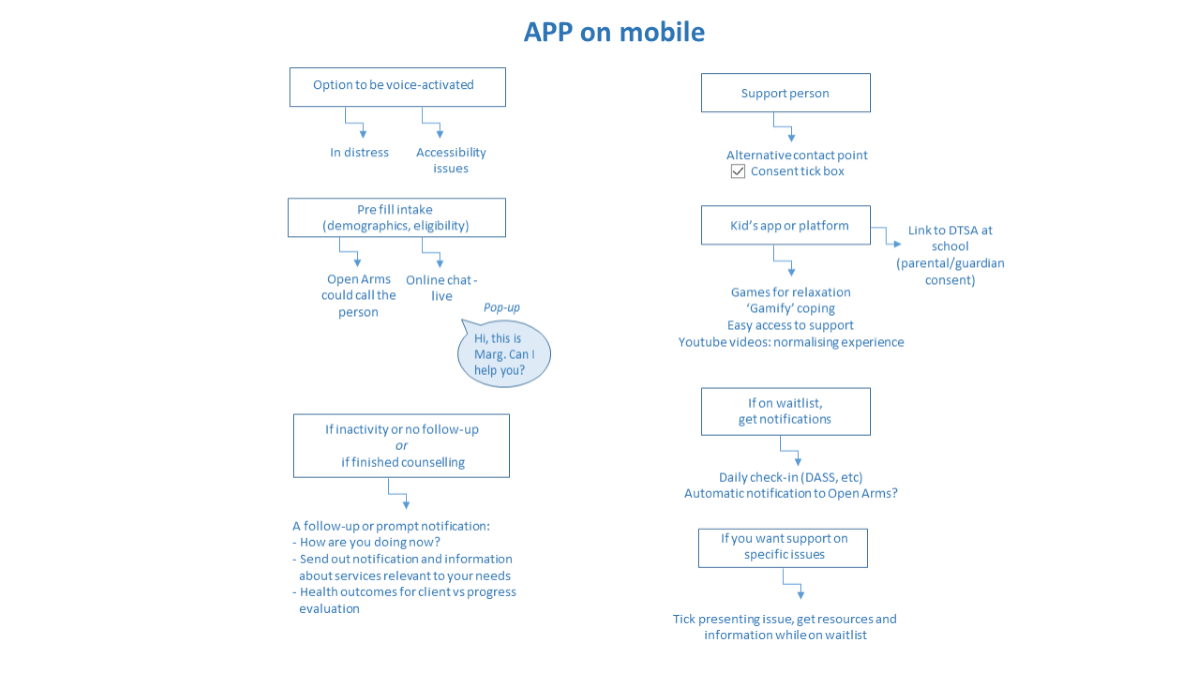
Sample wish list by participants for the technology.

In addition, participants suggested that the prototype could check-in with individuals routinely to ensure they were safe while waiting for care (Figure 3).

#### Progress Monitoring

Participants valued the potential for the prototype to offer progress monitoring to assess and prompt discussions about treatment effectiveness and provide individuals with feedback about their progress:

Progress monitoring is important to explore the effectiveness of treatments so changes in approach can be made as needed as well as to evaluate client satisfaction. If the client is achieving goals, this can also be reinforced.

While data tracking was indicated to be an important element of [the prototype] as a means to show progress, it was indicated that this may be alarming to clients showing a downward trajectory. It was suggested that [the prototype] might flag an alert in the platform for the service or primary health professional to check in with the client.

This feature is very helpful as it allows health professionals to make connections between a client’s dashboard as well as visualise a client’s progress.

The [prototype] should provide the individual feedback on their progress. The individual should have a sense that the [prototype] remembers them over time.

In addition, participants highlighted the potential for the prototype to check-in with clients for numerous reasons (Figure 3), which are as follows: (1) a period of inactivity or disengagement from the prototype, (2) progress monitoring in relation to health outcomes, and (3) notifications regarding relevant service offerings.

### Feedback on a Prototype for Immediate Risk Response, Known as the Need Help Now Button

**Figure 4:**
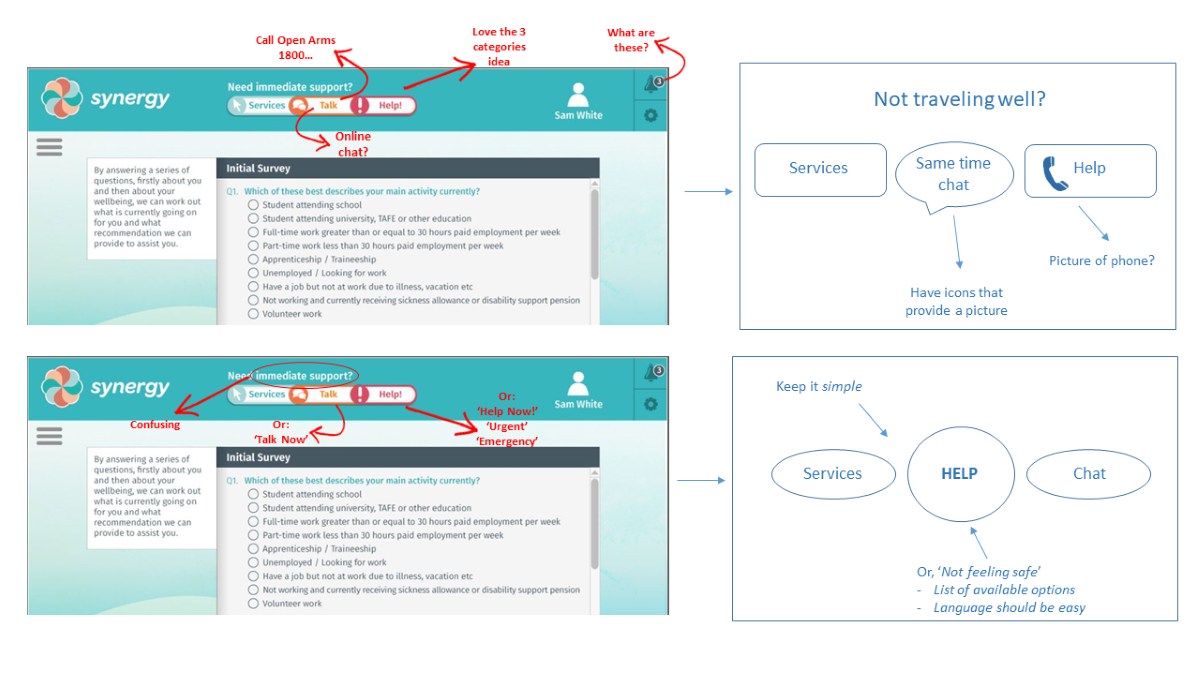
Combined feedback on Need Help Now button: annotated wireframes and suggested alternatives.

Given the call for improved risk detection and suicide prevention in the recent Senate inquiries [4], feedback on the *Need Help Now* button is particularly relevant. As pictured (Figure 4), the prototype included a 3-tiered support feature [18], including a list of geolocated mental health services, a Web-based chat feature, and a list of contact details for emergency services (eg, Triple 000, Lifeline, etc). Participants appreciated the tiered approach to risk response and provided suggestions for how this aspect of the technology could be improved upon, including personalization of the resources by an individual:

It is good that different options/ levels of care are presented.

Include the ability to list a buddy or partner here to be contacted in an emergency (ie, build your own “Need Help Now” button).

If a client does not like any of the resources that are included in the “Need Help Now” section, there needs to be an “other” option. This may be a phone service, a website, or an option to call a friend. You cannot leave people without an alternate option.

“Less is more.” If you are in crisis, there cannot be too many resources from which to choose. The selection process must be simple and clear.

Participants emphasized the need to ensure language in this feature is client centered:

The language used to label the buttons should be focused on what the client is experiencing or may be able to relate to (eg, not feeling safe).

“Emergency” is a more indicative term than “help.”

Participants provided specific comments on the *Need Help Now* prototype through annotating wireframes and by suggesting alternative designs (Figure 4).

### Technical Functionality

#### Data Security and Confidentiality

Integral to the design of any Web-based technology is the security of the data stored within the prototype. Within the context of Open Arms, participants consistently highlighted the need to ensure that their health information remains confidential and is not shared, for example, with other government organizations such as DVA, except with their express permission:

The terms and conditions and limitations to confidentiality need to be clearly explained.

Concerns about data confidentiality, including storage, access, ownership and accountability, will directly impact on how much the [prototype] is trusted.

Who has access to the data? How is it stored? Permissions need to be modifiable by the participant.

## Discussion

### User Engagement Through Participatory Design

Through the use of participatory design methodologies, members of the Open Arms community (including current and former ADF personnel and their family members, health professionals, service managers, and administrators) were actively engaged in the investigation of how to develop and configure a prototypic Web-based platform for Open Arms with the aim of enhancing the quality of the Open Arms service. A total of 7 participatory design workshops were conducted with a mix of members from the Open Arms community, as well as other key stakeholders, including representatives from the Australian Government (ie, DOH, DVA and Open Arms) and Project Synergy R&D and Product Groups, resulting in a wealth of information regarding current service practices and how to approach the co-design process for the Open Arms service.

Consistent with previous research, our findings highlight the value of engaging potential end users in the co-design process of new technologies [11,14]. Furthermore, the results of this study align with our previous experience working collaboratively with different stakeholders to increase access to and the effectiveness of mental health services for young people [17]. A review of the aggregate qualitative data (eg, feedback on wireframes, descriptive artifacts, and group-based development of user journeys) revealed that the prototype could provide the opportunity for greater and better-informed personal choice in relation to options for care based on level of need and personal preferences. To ensure transparency in care, the reconfigured prototype for Open Arms could allow individuals to have complete access to all of their individual health data presented in plain language as opposed to clinical jargon. Furthermore, the client would also have the option to share this information securely via the prototype with current and future health professionals to facilitate shared treatment planning, collaborative care and care continuity within and between services, and reduce the need for clients to repeat their clinical history or *story*.

The principles of the prototype align with the objectives set forth by DVA and Open Arms following the 2017 Senate inquiry [6,19]. In addition, participants identified several ways in which the prototype could enhance the Open Arms service pathway. In relation to intake, it was noted that a Web-based intake process could serve to fast-track individuals based on level of need and facilitate allocation of individuals to health professionals with the expertise matched to the identified mental health concerns. The potential benefits of progress monitoring over the course of care was also highlighted, particularly as a means by which to track and evaluate treatment effectiveness and provide active feedback to individuals and health professionals about progress and/or deterioration.

Importantly, the participatory design work completed with Open Arms has resulted in immediate service pathway redesign, highlighting the value of stakeholder engagement in research as a means to facilitate service-level changes [13]. Specifically, Open Arms has designed and implemented a revised intake process with the aim of reducing the time between initial service contact and the first appointment with a health professional. The previous intake process typically included 3 points of telephone contact (ie, pre-enrollment, intake, and allocation or appointment scheduling), whereas potentially only 2 points of contact will be required with the new process. Individuals, who are eligible for care through Open Arms, now contact a National Intake Service and have immediate access to an intake clinician, thus streamlining allocation to a health professional.

Notably, the research questions explored in this study are not unique to Open Arms but rather reflect universal themes related to technology-enabled mental health services reform. The configurable and customizable prototype will continue to be co-designed with input from individuals with lived experience, health professionals, and service staff (including administration and management) to support the prevention, early intervention, treatment, and continuous monitoring of mental ill-health and maintenance of well-being in people aged 2 years and older, including members of the veteran community [20]. Therefore, although Open Arms may develop a particular solution, such as a Web-based intake process specific to their service, the general concept of improved access to care is important universally, and thus will be explored with different groups to validate the thinking and further evolve the solution for broader use and applicability.

Although the co-design process is invaluable for the purposes of stakeholder engagement, the findings from this study are limited by their breadth. Further stakeholder engagement is now required to inform the co-design process of technology-enabled solutions aimed at addressing the critical challenges identified by Open Arms, including prevention and early identification, fast-tracking support, and capacity to provide the right care, first time.

End-user involvement in the co-design process is fundamental for the production of a relevant and usable technology solution [21,22]; however, the role of stakeholders must not end here. Existing literature highlights the necessity of establishing a feedback process to ensure the opportunity for critical assessment and iterative redesign of the solution [23]. As such, user testing is required to ensure the acceptance of the technology-enabled solution before implementation in the service as well as to facilitate the iterative redevelopment of the solution to adapt to the changing needs of the service and its consumers. This ongoing collaborative process of engagement will help ensure that members of the Open Arms community directly inform the development and configuration of the technology solution to help remove any barriers to help seeking, intake, assessment and treatment planning; enhance service delivery; and help improve the mental health and well-being of all individuals within the Open Arms community.

### Future Directions

An evaluation study to determine how new and emerging technologies could be used to enhance the Open Arms Sydney Centre, including safety and clinical quality, was approved by the Departments of Defence and Veterans’ Affairs Human Research Ethics Committee (DDVA HREC; project number: 017-17) and commenced in May 2018. This study allows for the continued active engagement of the Open Arms community through novel and innovative technologies, including service mapping, participatory design, and user testing. In addition, an impact evaluation research study has also been approved by DDVA HREC (project number: 056-18) and commenced in November 2018 with the aim of evaluating the quality of the revised intake process compared with the previous procedure. The primary objective will be achieved through an audit of service metrics, including time to first appointment, as well as potential differences between the phone-based intake process and an alternative offered by the prototype. Outcomes will include improved identification of individuals at risk of suicide. Allocation of clinical service and intervention intensity relative to an individual’s level of need (as identified using the clinical staging model at intake) will also be evaluated.

### Conclusions

Active engagement of individuals from the Open Arms community via participatory design methods demonstrated that the principles of the prototypic Web-based platform align with the recommendations made by DVA and Open Arms in response to 2017 Senate Inquiry report [6,19]. The breadth and depth of the information gathered through stakeholder engagement will now guide the development and configuration process of the technology-enabled solution for the Open Arms community, with the aim of ensuring high-quality, cost-effective, evidenced-based, person-centered mental health services for current and former ADF personnel and their family members.

## Abbreviations

ADF: Australian Defence Force
DDVA HREC: Departments of Defence and Veterans’ Affairs Human Research Ethics Committee
DOH: Department of Health
DVA: Department of Veterans’ Affairs
OPCs: Outreach Program Counselors
R&D: Research and Development
